# Carbon Monoxide in an Experimental Model of Chronic Pelvic Pain Syndrome: The Effects of CORM-A1 on Pain and Anxiety-Related Behaviors

**DOI:** 10.3390/pathophysiology32040053

**Published:** 2025-10-01

**Authors:** Nikola Šutulović, Neriman Ezgin, Nela Puškaš, Emilija Đurić, Željko Grubač, Daniel Škrijelj, Milena Vesković, Dušan Mladenović, Isidora Savić, Djuro Macut, Yavuz Dodurga, Aleksandra Rašić-Marković, Olivera Stanojlović, Dragan Hrnčić

**Affiliations:** 1 Institute of Medical Physiology “Richard Burian”, Belgrade University Faculty of Medicine, 11000 Belgrade, Serbia; 2 Department of Biotechnology, Institute of Natural and Applied Sciences, Cukurova University, Adana 01330, Turkey; 3 Institute of Histology and Embryology “Aleksandar Đ. Kostić”, Belgrade University Faculty of Medicine, 11000 Belgrade, Serbia; 4 Clinic of Urology, University Clinical Center of Serbia, Belgrade University Faculty of Medicine, 11000 Belgrade, Serbia; 5 Institute of Pathophysiology “Ljubodrag Buba Mihailovic”, Belgrade University Faculty of Medicine, 11000 Belgrade, Serbia; 6 Clinic of Endocrinology, Diabetes and Metabolic Disease, University Clinical Centre of Serbia, Belgrade University Faculty of Medicine, 11000 Belgrade, Serbia; 7 Department of Medical Biology, Faculty of Medicine, University of Pamukkale, Denizli 20160, Turkey

**Keywords:** prostatitis, anxiety-related behavior, carbon monoxide-realizing molecules, CORM A1, pain, rats

## Abstract

Current standard treatments for chronic prostatitis/chronic pelvic pain syndrome (CP/CPPS), a urological disorder with anxiety as a major comorbidity, are limited in success rates. Recent findings revealed the anti-inflammatory and neuroprotective effects of CO-releasing molecules (CO-RMs), but there is a gap in the knowledge on its effects in CP/CPPS. Therefore, the objective of our study was to investigate the potential therapeutic effects of CORM-A1 on the scrotal pain threshold and anxiety-related behaviors in experimental model of CP/CPPS. Adult Wistar albino male rats were randomized to Sham (intraprostatic saline) or CP/CPPS (intraprostatic λ-carrageenan) groups (*n* = 12). Half received CORM-A1 (2 mg/kg/day, i.p., days 1–7), others PBS, forming four subgroups (*n* = 6). The pain threshold (by an electronic von Frey esthesiometer) and anxiety-like behavior (by an open field, elevated plus maze and light/dark test) were assessed; prostates were histologically examined. Carrageenan-induced CP/CPPS caused significant mechanical pain hypersensitivity (*p* < 0.001), anxiety-like behaviors (*p* < 0.001–0.05), and histological prostate damage when compared to corresponding Sham groups. CORM-A1 treatment increased pain thresholds (*p* < 0.001) and improved behavioral outcomes (*p* < 0.001–0.01) in all ethological tests. These findings indicate that CORM-A1 exerts analgesic and anxiolytic effects in an experimental model of CP/CPPS in rats.

## 1. Introduction

Chronic prostatitis/chronic pelvic pain syndrome (CP/CPPS) is a complex and prevalent urological condition characterized by pelvic pain, embarrassment associated with urinary symptoms, and sexual dysfunction, as well as psychological comorbidities related to mental health, primarily including anxiety and depression [1,2]. With an incidence ranging up to 25% [3,4], CP/CPPS are among the most common urinary tract diseases. Despite this fact, its pathogenesis remains poorly understood.

With strong psychological components, CP/CPPS has been considered as a multifactorial and systemic syndrome, rather than just a prostate-specific disorder [5,6]. Quality of life in CP/CPPS patients is significantly debilitated by anxiety and depression [7,8]. Namely, it is reported that approximately 62% of patients with CP/CPPS demonstrate anxiety symptoms and high stress levels [9,10].

Mental health comorbidities seen in CP/CPPS patients seem to be closely related to pain-evoked hypothalamic–pituitary–adrenal (HPA) axis dysfunction, oxidative stress, and chronic (neuro)inflammation as key contributors to CP/CPPS pathophysiology [11,12]. Current standard treatments for CP/CPPS, such as anti-inflammatory drugs, alpha-blockers, and psychotropics, are both limited in their success rates and may cause significant side effects [13]. Therefore, novel, multifactorial treatment options targeting the neuroinflammatory and psychological components of CP/CPPS are needed.

Findings that the gasotransmitter carbon monoxide (CO) can modulate HPA axis activity [14], redox status [15,16], and inflammatory response [17] draw attention to CO-mediated signaling as a potential therapeutic target in mental health disorders [14,18], especially those associated with CP/CPPS. CO stands out as an innovative approach since it offers a different mechanism of action than classical pharmacological agents.

Carbon monoxide (CO) is endogenously generated via heme oxygenase enzymes (HO-1, HO-2), functioning as a gasotransmitter with anti-inflammatory, antiapoptotic, and cytoprotective effects [19]. To exploit these benefits while avoiding the hazards of inhaled CO, synthetic CO-releasing molecules (CO-RMs) were developed [20]. Many CO-RMs are transition metal carbonyl complexes (e.g., ruthenium, manganese, iron) that liberate CO spontaneously, through ligand exchange, interaction with biological species, or upon external triggers [21]. Non-metallic CO-RMs (e.g., CORM-A1, boranocarboxylates) provide alternative donor systems with lower toxicity and tunable release kinetics [21,22]. Structurally, CO-RMs typically consist of a metal or donor moiety coordinated with coligands, which regulate solubility, stability, and pharmacokinetics [23]. Their defining properties include controlled CO release, limited carboxyhemoglobin formation, and the potential for tissue-targeted therapeutic action, supporting their exploration as novel pharmacological tools [24].

CO-releasing molecules (CO-RMs) are recently developed chemicals that enable the controlled delivery of CO to tissues and organs [25]. It is reported that CO-RMs have the potential to exhibit neuroprotective effects against inflammation-induced brain damage [26,27]. According to the study by Luo et al. [28], CORM-A1 was found to have antidepressant and anxiolytic effects both at the cellular level and in animal models. However, the role of CO donors and the therapeutic potential of CORM-A1 in the pathology of CP/CPPS, where chronic inflammation and anxiety are seen together, remains unexplored.

Experimental models of CP/CPPS are essential for studying the underlying mechanisms and testing novel therapeutics. Several approaches have been described, including bacterial infection models, autoimmune prostatitis models, hormone imbalance models, and chemical or mechanical injury-induced inflammation [29,30,31]. Among these, the intraprostatic injection of λ-carrageenan has gained prominence because it produces reproducible pelvic pain, local inflammation, and behavioral alterations resembling clinical CP/CPPS [32,33,34,35]. This model is characterized by persistent nociception, leukocyte infiltration, and proinflammatory mediator upregulation, accompanied by anxiety-like behavior in rodents [32,36]. Its reliability and translational relevance make it particularly suitable for evaluating both analgesic and anxiolytic therapeutic strategies, as employed in the present study.

This study aimed to address this gap by investigating the effects of system CORM-A1 on pain sensitivity and anxiety-like behavior in the CP/CPPS animal model. Our objectives were to assess the effects of CORM-A1 on the mechanical scrotal pain threshold and behavior of rats with carrageenan-induced CP/CPPS in open field (OF), elevated plus maze (EPM) and light/dark test (L/D) tests.

## 2. Materials and Methods

### 2.1. Animals and Ethical Statements

The study used Wistar albino male rats weighing between 250 and 300 g (*n* = 24). The animals were housed under standard laboratory conditions, including constant temperature (22 ± 2 °C) and relative air humidity (50 ± 5%), a 12 h light/dark cycle (light starting at 08:00 a.m.), and free access to water and food.

All procedures conducted in this study received approval from the Ethics Committee of the University of Belgrade and were carried out in compliance with the international guidelines and in agreement with the Local Ethics Committee for Animal Research. (Permission No. 323-07-01339/2017-05/3).

### 2.2. Study Design

In this study, rats were assigned randomly into two primary groups: the Sham group (*n* = 12, intraprostatic 0.9% NaCl injection at time of surgery) or the CP/CPPS group (*n* = 12, intraprostatic 3% λ-carrageenan injection).

Afterwards, half of the animals for both groups were administered CORM-A1 (sodium boranocarbonate, 2 mg/kg/day, i.p. dissolved in PBS and administered in a volume of 0.1 mL/kg from the first to the seventh day after surgery), while the other half were given phosphate-buffered saline (PBS, 0.1 mL/kg/day, i.p,) as the solvent. Thus, the following four groups were established (*n* = 6 in each): 1: Sham-PBS, 2: Sham-CORM, 3: CP/CPPS-PBS, 4: CP/CPPS-CORM. CORM-A1 dosage was selected based on previous in vivo studies in rodents [16,37,38].

**Figure 1:**
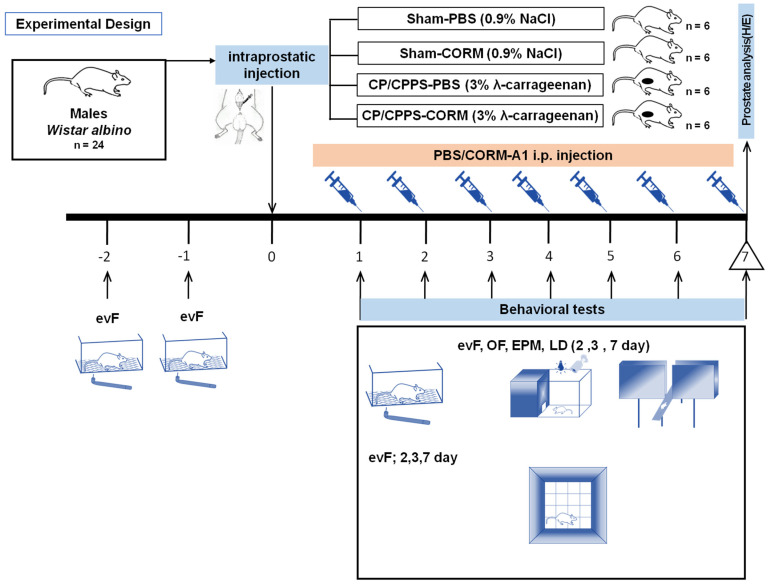
Adult male Wistar albino rats (*n* = 24) were subjected to an adaptation process from the ninth (−9) day to the second (−2) day before surgery. eVF (electronic von Frey) adaptation to evaluate the mechanical pain threshold in the scrotal region was performed two (−2) and one (−1) days before surgery. Rats were randomly divided into four groups according to the type of intraprostatic injection during surgery (day 0) and postoperative treatment: Sham-PBS (intraprostatic 0.9% NaCl injection and daily postoperative PBS administration, 0.1 mL/kg; *n* = 6); Sham-CORM (intraprostatic 0.9% NaCl injection and daily postoperative CORM-A1 administration, 2 mg/kg; *n* = 6); CP/CPPS-PBS (intraprostatic 3% λ-carrageenan injection and daily postoperative PBS administration, 0.1 mL/kg; *n* = 6); CP/CPPS-CORM (intraprostatic 3% λ-carrageenan injection and daily postoperative CORM-A1 administration, 2 mg/kg; *n* = 6). Mechanical pain sensitivity in the scrotal region was evaluated by the evF device and anxiety-related behaviors were assessed by light/dark (L/D), open field (OF), and elevated plus maze (EPM) tests. On postoperative day 7, rats were sacrificed after behavioral testing; prostates were sampled and analyzed by hematoxylin–eosin staining.

The mechanical pain threshold was evaluated using an electronic von Frey esthesiometer (evF), and anxiety-related behaviors were assessed by light/dark (L/D), open field (OF), and elevated plus maze (EPM) tests on the second (2nd), third (3rd), and seventh (7th) postoperative days. On postoperative day 7, the rats were sacrificed after behavioral testing; prostates were sampled and analyzed by hematoxylin–eosin staining (Figure 1). Researchers were blinded to the treatment group during the behavioral testing and histological evaluation.

### 2.3. Establishment of the Experimental CP/CPPS Model and Surgical Operation

The CP/CPPS model was established in this study according to already reported protocols [32,33]. Briefly, the surgical and treatment procedure was as follows: sodium thiopental (40 mg/kg) was administered intraperitoneally to the rats to provide general anesthesia. Then, the scrotum and lower abdomen were depilated, and the animals were fixed supine on a heating pad to maintain body temperature. After the surgical site was disinfected, a subcutaneous local anesthetic was applied to the incision line. A vertical incision (1–1.5 cm) was carefully made in the lower abdominal wall along the midline, and the ventral prostate lobes were exposed. The animals in the CP/CPPS group were injected with 25 μL of sterile 3% λ-carrageenan solution into the left and right prostate lobes using a sterile Hamilton^®^ syringe. The animals in the Sham group were given 0.9% NaCl by the same method. Once the intraprostatic injection was completed, local anesthetic was reapplied at the incision site, and the surgical opening was sutured with absorbable material (4-0 Polysorb™). The animals that awoke from anesthesia were placed in their cages for observation and recovery.

### 2.4. Evaluation of Mechanical Pain Sensitivity: Scrotal Pain Threshold

To monitor the development of experimental CP/CPPS and evaluate mechanical hyperalgesia/ allodynia, the scrotal pain threshold was measured on the 1st and 2nd day before the intraprostatic procedure and on the 2nd, 3rd, and 7th day after the postoperative day. Measurements were made using an electronic von eVF. Animals were subjected to adaptation in Plexiglas chambers twice daily for 15–20 min before measurement and for 30 min during measurement. The eVF device was placed in contact with the scrotal skin perpendicularly and the pressure gradually increased. When the withdrawal reflex response was obtained, the maximum pressure value on the device screen was recorded. The pain threshold was defined as the value of the lowest mechanical force that produced a reflex response and calculated as the average of three measurements. After the measurements, the animals were placed in their cages.

### 2.5. Behavioral Tests

#### 2.5.1. Open Field Test

The OF test was carried out using a soundproof chamber equipped with infrared sensors (Experimetria Ltd., Budapest, Hungary) and software designed for automated analysis (Conducta System v 1.0, Experimetria Ltd., Budapest, Hungary) [35].

The test area was designed as an open area surrounded by black walls and illuminated with red light. The rats were placed individually in this area and their horizontal and vertical locomotor activities were recorded for 15 min. The measured parameters included total distance move (cm), total walking time (s), and the number of rearing movements. To assess the spatial distribution of locomotor activity, the test area was virtually divided into 16 squares, 4 of which were designated as the center of the area. The duration the animal remained in the central zone and the thigmotaxis index were calculated. The thigmotaxis index was determined as the ratio of distance made in the peripheral areas to the total distance and expressed as a percentage. Decrease in time that the animal spent in the central zone, decrease in number of rearing movements, and a higher thigmotaxis index are widely accepted indicators of anxiety-like behavior [35].

#### 2.5.2. Elevated Plus Maze Test

The EPM was performed according to the protocol described in [39,40]. Briefly, the EPM platform consisted of four arms placed at a height of 50 cm and connected at right angles. Each arm was 50 × 10 cm in size and there was a central platform measuring 10 × 10 cm in the middle (Elunit, Belgrade, Serbia). The EPM platform was observed via an infrared monitoring system (HikVision Bullet 2612, Hangzhou, China) for 5 min upon placing the animal in the central area. The maze was cleaned with 70% ethanol between each animal test to remove olfactory cues. Afterwards, the animal’s behavior was analyzed by an independent researcher blinded to the treatment. The transitions between open and closed arms and the time spent in the closed arms were recorded and analyzed to determine the anxiety level. Decrease in number of transitions between open and closed arms and increase in time spent in the closed arms indicate a higher anxiety level in animals [39,40].

#### 2.5.3. Light/Dark Test

A two-chamber system was used for the light/dark behavior test [41]. The test setup consisted of a light and a dark compartment. The light compartment was white, while all surfaces of the dark compartment were black, and the top was covered with an opaque lid. The transition between the two compartments was provided by a square opening. The top of the light compartment was left open and a video camera (Logitech C210, Lausanne, Switzerland) was placed there and the recording was performed. The animals were positioned centrally within the light compartment at the beginning of the test, and their free behavior was recorded for 5 min. The key parameters evaluated in this test were the number of transitions between compartments and the total time spent in the light compartment. Increase in the number of transitions between compartments and decrease in the total time spent in the light compartment are indicators of a higher anxiety level in animals [41]. Behavioral analyses were performed by an investigator blinded to the experimental protocol.

### 2.6. Histological Analysis of the Prostate

For histological analysis of the prostate, dissection was performed after the rats were sacrificed by decapitation and prostate tissues were fixed in 10% buffered formalin for 24 h. After fixation, prostate samples were dehydrated in increasing alcohol concentrations (70%, 96%, 100%), cleared in xylene and embedded in paraffin. The samples were sectioned at 5 μm thickness. Routine hematoxylin–eosin staining was performed to examine inflammation. The histological slides were examined under a light microscope, and representative photomicrographs were taken.

### 2.7. Substances

All substances used in this study (λ-carrageenan, PBS, CORM-A1) were a product of Sigma Aldrich, St. Louis, MO, USA, and were of analytical grade. All solutions were freshly prepared before the administration.

### 2.8. Data Processing

The Kolmogorov–Smirnov test was used to test the normality of the data. In the study, data that followed a normal distribution are presented as mean ± standard deviation (SD). One way ANOVA was applied to test the effects between groups. For the analysis of within-group differences at different time points, repeated measures ANOVA was used. Tukey–Kramer LSD was used as a post hoc test. Statistical significance was assessed as *p* < 0.001, *p* < 0.01, and *p* < 0.05.

## 3. Results

### 3.1. Effect of CORM-A 1 on Scrotal Pain Threshold

**Figure 2:**
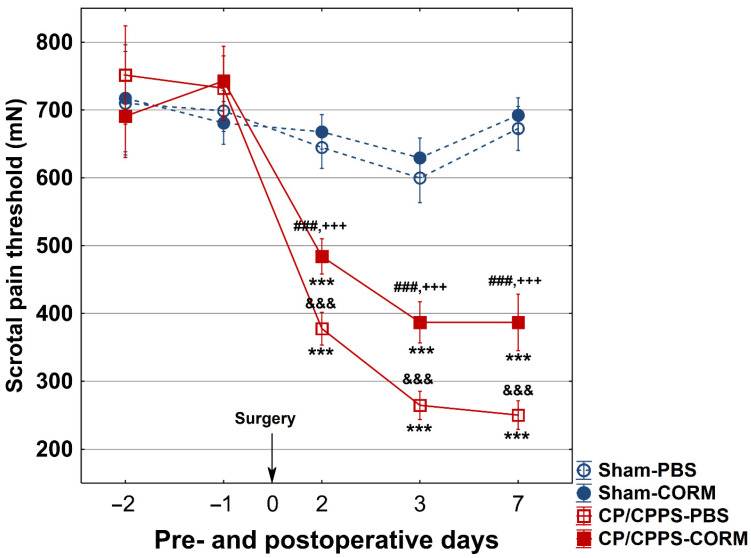
Effect of CORM-A1 on the scrotal pain threshold in animals with CP/CPPS. Animals underwent scrotal pain threshold testing with an eVF esthesiometer 2 and 1 days before surgery and on the 2nd, 3rd, and 7th postoperative day. Animals were divided into two groups according to intraprostatic injections: Sham (0.9% NaCl) and CP/CPPS (3% λ-carrageenan). These two groups were treated with PBS or CORM-A1 for 7 postoperative days, creating four groups: Sham-PBS, Sham-CORM, CP/CPPS-PBS, and CP/CPPS-CORM. Data are presented as mean ± standard deviation. Differences between groups were assessed using one-way ANOVA and Tukey–Kramer LSD post hoc test (&&& *p* < 0.001 vs. Sham-PBS; +++ *p* < 0.001 vs. Sham-CORM; ### *p* < 0.001 vs. CP/CPPS-PBS). Intra-group comparisons were made using the repeated measures ANOVA (*** *p* < 0.001, vs. −1). For details, see Figure 1.

In all groups (Sham-PBS, Sham-CORM, CP/CPPS-PBS, and CP/CPPS-CORM), the scrotal pain threshold was measured before (1st and 2nd day) and after (2nd, 3rd, and 7th day) surgery. There was no significant difference between the groups at baseline (*p* > 0.05, Figure 2). In the Sham groups (PBS and CORM), the pain threshold did not change before and after surgery (*p* > 0.05). However, in the CP/CPPS groups (with 3% λ-carrageenan injection), the pain threshold decreased significantly on the 2nd, 3rd, and 7th postoperative days (*p* < 0.001). This decrease was significant both compared to the Sham groups and their own preoperative values. Importantly, CORM-A1 administration significantly increased the pain threshold in the CP/CPPS-CORM group and provided lower pain levels than the CP/CPPS-PBS group (*p* < 0.001 for all postoperative days). This shows that CORM-A1 has a pain-relieving effect. In the Sham groups, CORM-A1 application did not cause a significant change in the pain threshold (*p* > 0.05, Figure 2).

### 3.2. Effect of CORM-A1 on Anxiety-Related Behavior

#### 3.2.1. Open Field Tests

**Figure 3:**
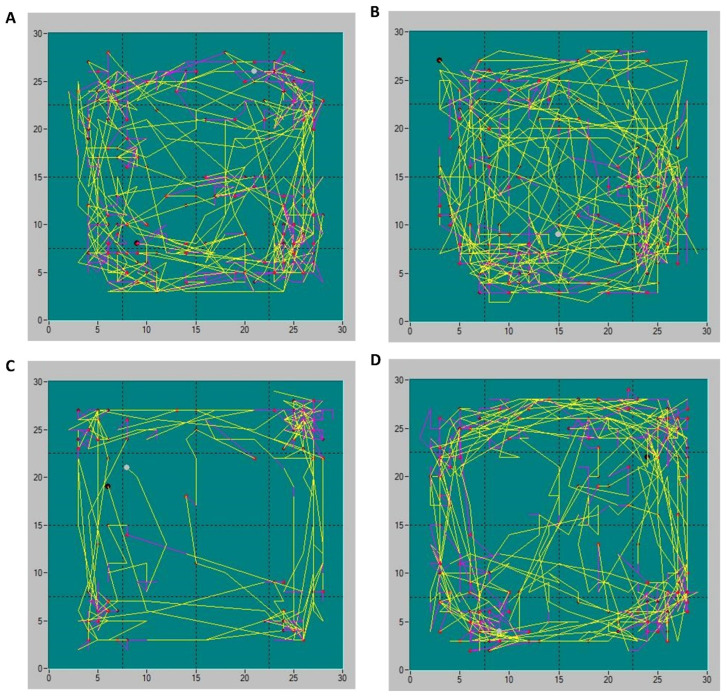
Representative trajectories of animals’ locomotor activities in Sham-PBS (**A**), Sham-CORM (**B**), CP/CPPS-PBS (**C**), and CP/CPPS-CORM (**D**) groups in the open field test. Each animal was subjected to the open field trial for 15 min. Different types of movements are depicted by different line colors: yellow-ambulatory movements, purple-vertical activity. Activity was recorded with an infrared sensor system and analyzed with Conducta 1.0 software. For a detailed explanation, see Figure 1 and Figure 2.

Recorded trajectories of locomotor activity showed visually distinct behavioral patterns between the groups. Representative images of the animals’ movement trajectories are presented in Figure 3.

**Figure 4:**
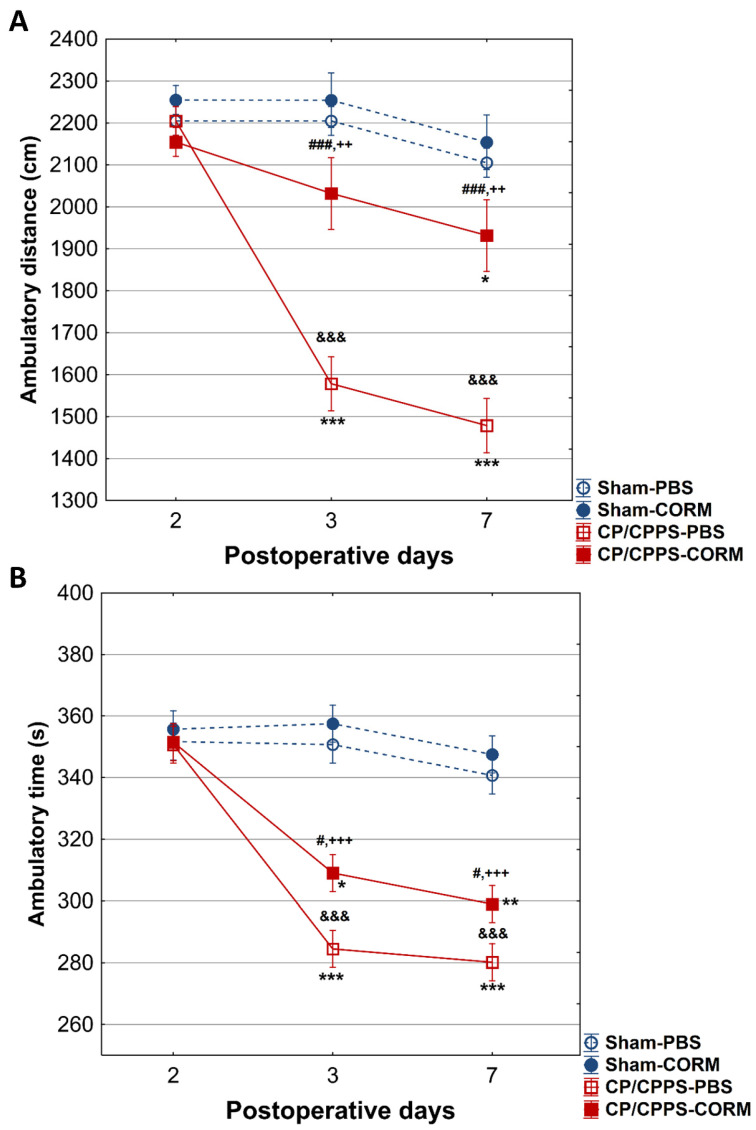
Effect of CORM-A1 on total distance (**A**) and on total ambulatory time (**B**) in the open field test in animals with CP/CPPS. Values are expressed as mean ± standard deviation. Statistical significance of differences in total distance between groups was determined using a one-way ANOVA test with Tukey–Kramer LSD post hoc testing (&&& *p* < 0.001, vs. Sham-PBS; ++ *p* < 0.01, vs. Sham-CORM; ### *p* < 0.001 vs. CP/CPPS-PBS), (&&& *p* < 0.001 vs. Sham-PBS; +++ *p* < 0.001 vs. Sham-CORM; # *p* < 0.05, vs. CP/CPPS-PBS). The statistical significance of the differences within each group was assessed using the same test (* *p* < 0.05, ** *p* < 0.01, *** *p* < 0.001, vs. 2). For a detailed explanation, see Figure 1 and Figure 2.

In the horizontal locomotor activity analysis, animals with prostatitis (CP/CPPS-CORM and CP/CPPS-PBS) on days 3 and 7 covered shorter distances and walking time compared to Sham groups (*p* < 0.001 and *p* < 0.01 Figure 4A,B). Similarly, animals with prostatitis treated with CORM-A1 had longer distances compared to those treated with PBS (*p* < 0.001). However, CP/CPPS-CORM animals significantly decreased total distance on postoperative day 7 compared to postoperative day 2 (*p* < 0.05, Figure 4A), while no significant difference was detected in the Sham groups (*p* > 0.05).

Importantly, intragroup analysis of total walking movement time in animals in the CP/CPPS-PBS group showed a statistically significant decrease on days 3 (*p* < 0.001) and 7 (*p* < 0.001) compared to postoperative day 2 (Figure 4B). In addition, total movement time in the CP/CPPS-CORM group decreased significantly on days 3 (*p* < 0.05) and 7 (*p* < 0.01) compared to postoperative day 2 (Figure 4B).

**Figure 5:**
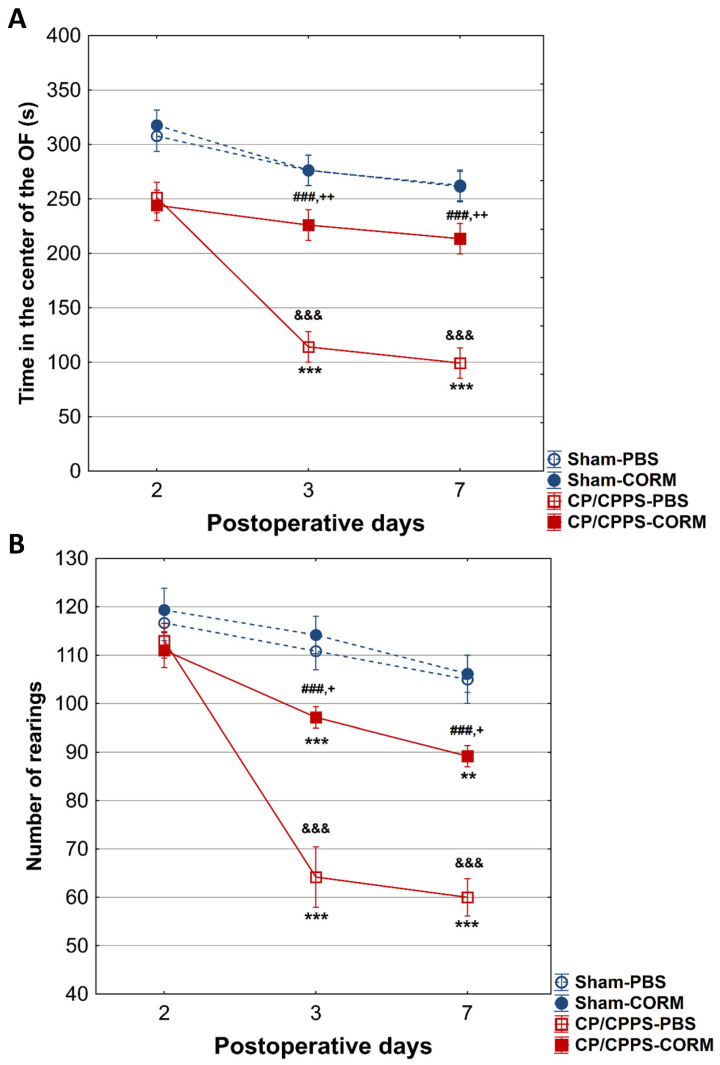
Effect of CORM-A1 on time spent in the center (**A**) and number of rearing movements (rearings, (**B**)) in the open field in animals with CP/CPPS. Values are expressed as mean ± standard deviation. The statistical significance of the between-group difference in time spent in the center of the open field was determined using a one-way ANOVA test with Tukey–Kramer LSD post hoc testing (&&& *p* < 0.001 vs. Sham-PBS; + *p* < 0.05 vs. Sham-CORM; ++ *p* < 0.01, vs. Sham-CORM; ### *p* < 0.001 vs. CP/CPPS-PBS). Statistical significance of differences within each group was assessed using the same test (** *p* < 0.01, *** *p* < 0.001, vs. 2). See the legend to Figure 1 for details.

On postoperative days 3 and 7, CP/CPPS animals spent less time in the center of the open field than the Sham groups (*p* < 0.001). However, CORM-A1 treatment significantly increased this time in CP/CPPS animals (*p* < 0.001 for both days). On the same days, animals in the CP/CPPS-CORM group exhibited more rearing movements than in the PBS group (*p* < 0.001; Figure 5B). No change was observed in the Sham groups (*p* > 0.05; Figure 5A,B).

**Figure 6:**
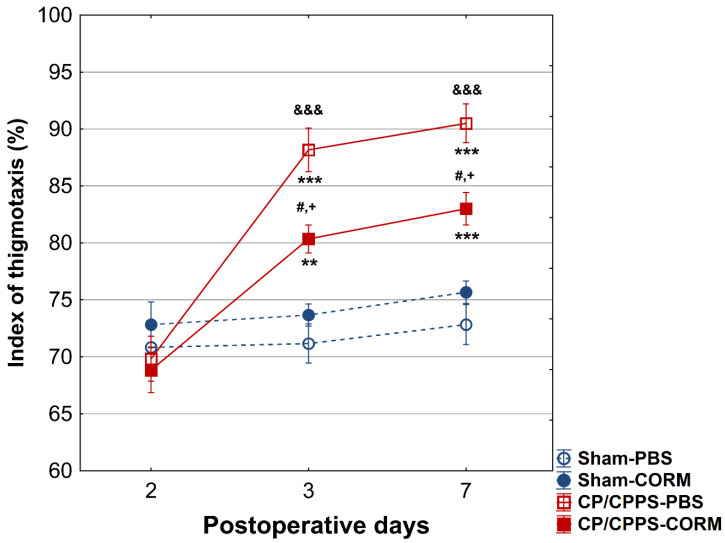
Effect of CORM-A1 on the thigmotaxis index in the open field test in animals with CP/CPPS. Values are expressed as mean values ± standard deviation. Statistical significance of the difference in the thigmotaxis index was determined using a one-way ANOVA test with a Tukey–Kramer LSD post hoc test (&&& *p* < 0.001 vs. Sham-PBS; + *p* < 0.05 vs. Sham-CORM; # *p* < 0.05 vs. CP/CPPS-PBS). Statistical significance of differences within each group was assessed using the same test (** *p* < 0.01, *** *p* < 0.001, vs. 2). For a detailed explanation, see Figure 1.

The thigmotaxis index was significantly higher in CP/CPPS animals compared to Sham groups on postoperative days 3 and 7, (*p* < 0.001 or *p* < 0.05). CORM-A1 treatment was effective in reducing thigmotaxis in prostatitis animals compared to the PBS group (*p* < 0.05 for both days). In addition, thigmotaxis were significantly increased in CP/CPPS-PBS group on days 3 and 7 compared to day 2 (*p* < 0.001; Figure 6).

#### 3.2.2. Elevated Plus Maze Test

**Figure 7:**
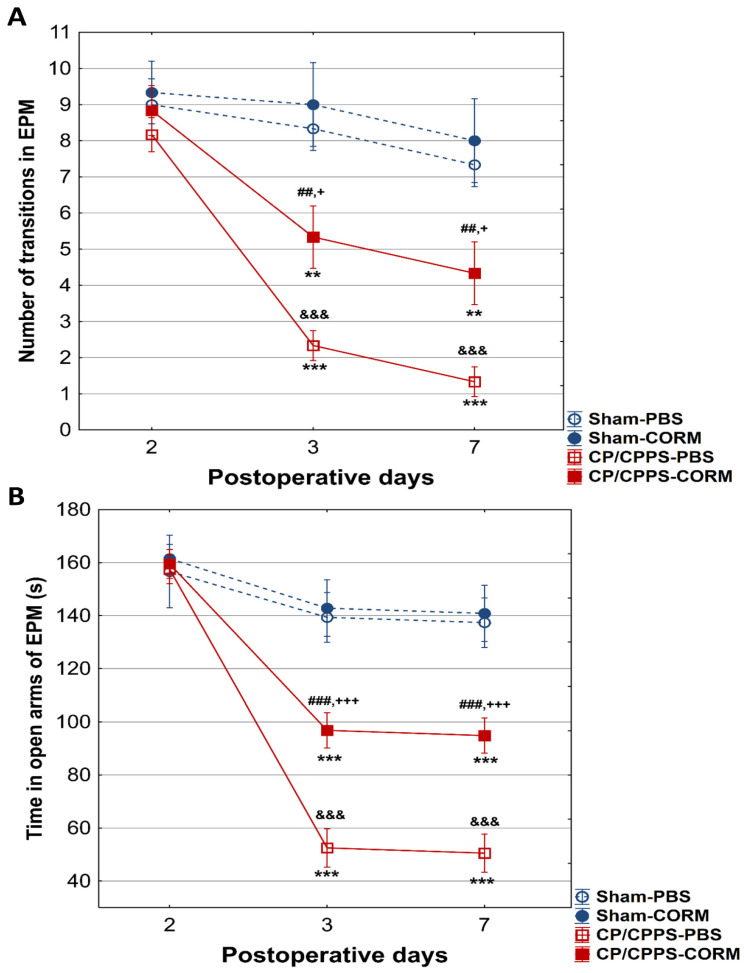
Effect of CORM-A1 on (**A**) the number of transitions and the time spent in the open arms (**B**) of the elevated plus maze in animals with CP/CPPS. &&& *p* < 0.001 vs. Sham-PBS; + *p* < 0.05 vs. Sham-CORM; +++ *p* < 0.001, vs. Sham-CORM; ## *p* < 0.01 vs. CP/CPPS-PBS; ### *p* < 0.001, vs. CP/CPPS-PBS, ** *p* < 0.01, *** *p* < 0.001, vs. 2. For a detailed explanation, see Figure 1 and Figure 2.

On the 2nd postoperative day, no differences were recorded between the groups in the number of transitions between the open and closed arms of the maze (*p* > 0.05, Figure 6). Also, no considerable difference was observed between the groups regarding the duration spent in the open arms on the 2nd postoperative day (*p* > 0.05, Figure 7B).

On the other hand, on the 3rd and 7th day, animals with prostatitis made significantly fewer transitions than the corresponding Sham groups (Sham-PBS and CP/CPPS-PBS *p* < 0.001; Sham-CORM and CP/CPPS-CORM *p* < 0.05, Figure 7A).

Moreover, on days 3 and 7, the duration spent in the open arms was also reduced in prostatitis-induced animals compared to the Sham groups (Sham-PBS and CP/CPPS-PBS *p* < 0.001; Sham-CORM and CP/CPPS-CORM *p* < 0.001, Figure 7B).

Importantly, CORM-A1-treated animals with prostatitis made significantly more transitions compared to PBS-treated animals with prostatitis on the 3rd (*p* < 0.001), as well on and 7th (*p* < 0.01) day (Figure 7A). The same holds true for time spent in open arms. Namely, animals with prostatitis treated with CORM-A1 spent significantly more time in open arms relative to those animals with prostatitis treated with PBS (days 3 and 7, CP/CPPS-CORM vs. CP/CPPS-PBS, *p* < 0.001, Figure 7B).

#### 3.2.3. Light/Dark Test

**Figure 8:**
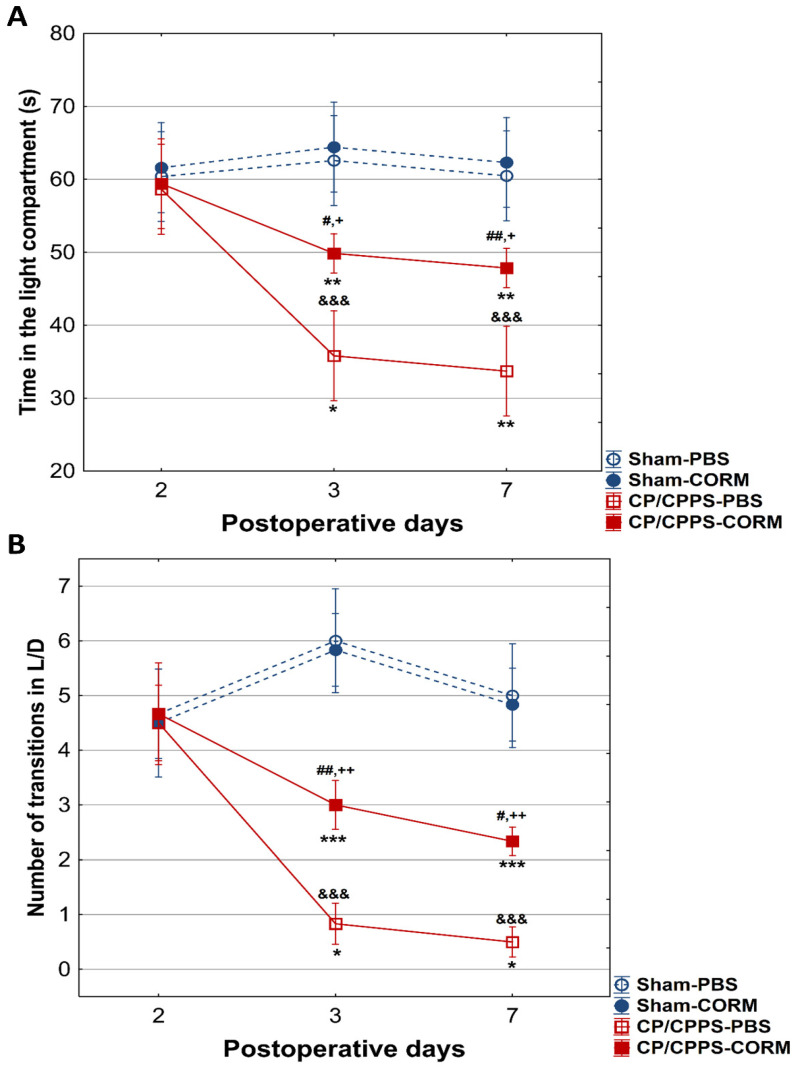
Effect of CORM-A1 on the time spent in the light compartment (**A**) and number of transitions from the light to the dark compartment (**B**) in CP/CPPS rats in the light/dark test. &&& *p* < 0.001 compared to Sham-PBS; + *p* < 0.05, ++ *p* < 0.01 compared to Sham-CORM; # *p* < 0.05, ## *p* < 0.01 compared to CP/CPPS-PBS; * *p* < 0.05, ** *p* < 0.01, *** *p* < 0.001 compared to day 2. For a detailed explanation, see Figure 1 and Figure 2.

In the L/D test, no statistically significant difference was observed between the groups regarding the time spent in the light compartment, as well as the number of transitions between compartments on the 2nd postoperative day (Figure 8A,B).

On days 3 and 7, a significant decline in the amount of time spent in the light compartment was recorded in the CP/CPPS-PBS and CP/CPPS-CORM groups relative to the control groups (Sham-PBS vs. CP/CPPS-PBS, *p* < 0.001; Sham-CORM vs. CP/CPPS-CORM *p* < 0.05; Sham-PBS vs. CP/CPPS-CORM *p* < 0.001; Figure 8A). The same trend was observed for the number of transitions. Namely, on days 3 and 7, animals with prostatitis made fewer transitions compared to corresponding sham controls (CP/CPPS-PBS vs. Sham-PBS, *p* < 0.001; Sham-CORM vs. CP/CPPS-CORM *p* < 0.01, Figure 8B). In addition, animals with prostatitis treated with CORM-A1 made more transitions than those treated with PBS on days 3 and 7 (*p* < 0.01). These findings indicate that CORM-A1 treatment decreases anxiety-related behaviors in animals with prostatitis.

### 3.3. Effect of CORM-A1 on the Histological Structure of the Prostate

**Figure 9:**
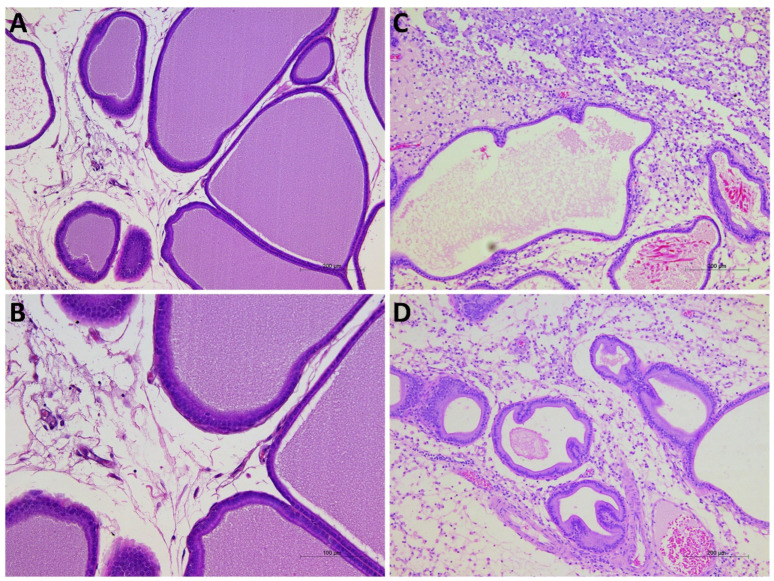
Representative photomicrographs of prostate tissues from Sham-PBS (**A**), Sham-CORM (**B**), CP/CPPS-PBS (**C**), and CP/CPPS-CORM (**D**) groups. While the prostate tissue maintained its normal structure in the Sham-PBS (**A**) and Sham-CORM (**B**) groups, significant interstitial proliferation, inflammatory infiltrate, hyalinization, necrosis, and vascular congestion were observed in the CP/CPPS-PBS group (**C**). In the CP/CPPS-CORM group (**D**), CORM-A1 treatment alleviated these findings; hyalinization was less pronounced, and necrosis was absent.

The histological examination of prostate tissues revealed no remarkable structural changes in the Sham groups (Sham-PBS and Sham-CORM groups treated with 0.9% NaCl), with preserved glandular architecture, intact epithelial lining, and absence of leukocyte infiltration (Figure 9A,B). In contrast, the CP/CPPS-PBS group demonstrated clear signs of inflammation, interstitial proliferation, hyalinization, necrosis, and vascular congestion including epithelial disruption, stromal edema, and prominent infiltration of inflammatory cells within the glandular stroma and perivascular regions. These alterations are consistent with the acute inflammatory response induced by intraprostatic λ-carrageenan injection (Figure 9C). On the other hand, in the CP/CPPS-CORM group, 7-day CORM-A1 treatment qualitatively reduced inflammatory cell infiltration and partially preserved epithelial integrity compared with CP/CPPS-PBS, suggesting a protective effect of CORM-A1 against carrageenan-induced prostatic injury; although proliferation and infiltration continued, hyalinization decreased, and necrosis was not observed (Figure 9D).

## 4. Discussion

In the current study, we investigated the effects of system CORM-A1 on pain sensitivity and anxiety-like behavior in the CP/CPPS animal model. Our objectives were to assess mechanical pain sensitivity by determining the scrotal pain threshold, evaluate anxiety-like behaviors using open field (OF), elevated plus maze (EPM), and light/dark test (L/D) tests, and examine prostate histopathological changes following system CORM- A1 treatment in rats with carrageen-induced CP/CPPS.

The results of our current study, for the first time, revealed that the systemic administration of CORM-A1 significantly ameliorates both mechanical allodynia and anxiety-like behaviors in the CP/CPPS rat model induced by 3% λ-carrageenan. Our findings emphasize the potential of CORMs as promising therapeutic options for chronic prostatic inflammation and its mental health-related comorbidities.

In this study, rats receiving vehicle upon CP/CPPS induction (CP/CPPS-PBS group) exhibited mechanical allodynia, a hallmark feature of CP/CPPS in humans. It was manifested as a decreased pain threshold to mechanical stimulation as recorded by evF (baseline vs. postoperative measurements, as well as Sham vs. CP/CPPS, Figure 2). The most profound decrease was observed 7 days upon 3% λ-carrageenan intraprostatic injection. On the other hand, the mechanical scrotal pain threshold has been significantly elevated in rats treated with CORM-A1 upon induction of CP/CPPS (CP/CPPS-CORM-A1 group) compared to corresponding controls receiving vehicle (CP/CPPS-PBS group; on the 2nd, 3rd, and 7th postoperative day, Figure 2). Thus, these findings indicate that exogenous CO ameliorated pain hypersensitivity provoked by CP/CPPS.

However, there are conflicting findings regarding the role of CO in pain perception. While some studies suggest that CO may increase allodynia and hyperalgesia by causing pronociceptive effects at the spinal cord level [42], others have reported that CO may have antinociceptive effects on the central structures involved in pain perception [43]. Moreover, it has been suggested that increased endogenous CO production, especially in inflamed tissues, may suppress pain perception [44]. In line with these findings, a recent study reported that CO and NO reduce the effects of peripheral inflammation by activating HO-1 pathways and increasing the expression of the μ-opioid receptor (MOR) and δ-opioid receptor (DOR) in the dorsal hippocampus, which modulate pain perception in the body and regulate responses to pain [45]. Additionally, very recent in vivo and in vitro studies showed that some novel CORMs could alleviate pain in a model of rheumatoid arthritis, and tendon remodeling in a model of tendinopathy by different mechanisms [46,47,48]. The increase in scrotal pain threshold observed in the CORM-A1-treated rats with CP/CPPS in our study is in agreement with these findings and may be related to the therapeutic effects of CO.

In our study, H&E histological evaluation of prostate tissue revealed that a 3% λ-carrageenan injection caused all histopathological signs of prostate inflammation, like interstitial proliferation, intense mononuclear infiltration, hyalinization, necrosis, and vascular congestion of significant tissue damage. These changes were not seen in the Sham groups. On the other hand, in rats treated with CORM-A1, upon λ-carrageenan intraprostatic injection (CP/CPPS-CORM group), we observed alleviated inflammation; although proliferation and infiltration continued, hyalinization decreased, and necrosis was not observed.

Huang et al. (2020) reported that psychological factors and pain catastrophizing thoughts are common in men with CP/CPPS and that this may be related to hyperalgesia [49]. Similarly, Stamatio et al. (2024) showed that there was a significant positive correlation between the anxiety level and the severity of prostatitis-like symptoms in CP/CPPS patients [2]. It has also been reported that stress and anxiety are effective factors in the onset, prolongation, and maintenance of CP/CPPS symptoms [50]. These findings suggest that there is a bidirectional relationship between CP/CPPS symptomatology and anxiety and that this interaction may negatively affect the clinical course of CP/CPPS. Our results suggest that carbon monoxide-releasing molecules may have the potential to break this vicious cycle.

In order to assess the anxiety-like behavior, we used a battery of ethological tests consisting of the OF, EMP, and LD test in this study. All rats with CPPS manifested increased anxiety-like behavior in these tests on the 3rd and 7th day upon intraprostatic 3% λ-carrageenan injection compared to corresponding Sham groups. These results corroborated previous findings demonstrating the existence of anxiety-like behavior in CP/CPPS rats [12,32,33]. Moreover, our previous study showed that increased anxiety-like behaviors in CP/CPPS rat models were closely associated with increased brain oxidative stress, increased serum corticosterone levels, and loss of parvalbumin-positive interneurons in the hippocampus [12]. These findings support the hypothesis that peripheral inflammation may affect the central nervous system through a series of biochemical and immunohistochemical changes, leading to both behavioral and mental disorders. Chao Hu et al. (2016) showed that prostate-derived cytokines crossed the blood–brain barrier and modulated ERK1/2 signaling pathways, especially in mood and memory centers such as the amygdala, nucleus accumbens, and the hippocampus, which may play a role in the occurrence of anxiety and other mental health disorders [51].

Results of herein applied ethological tests demonstrated anxiolytic-like effects of systemic CORM-A1 administration in rats with experimentally induced CP/CPPS. Namely, postoperative CORM-A1 treatment of CP/CPPS rats led to a reversion of anxiety-like behavior in comparison to the vehicle-treated rats consistently in all these behavioral tests.

In the OF test, CORM-A1-treated CPPS rats spend significantly more time in the center compared to corresponding controls receiving vehicle (CPPS-PBS, on the 3rd and 7th postoperative day, but not on the 2nd postoperative day (Figure 5)). At the same time, the number of rearing movements was increased, and the index of thigmotaxis decreased in CP/CPPS rats treated with CORM-A1 compared to those treated with vehicle. According to this pattern of activity in the OF, CORM-A1 reversed anxiety-like behavior.

In the EPM test, CORM-A1-treated animals with CP/CPPS made significantly more transitions from open to closed arms compared to vehicle-treated animals with CP/CPPS on the 3rd, as well on the 7th postoperative day. The same holds true for time spent in the open arms (Figure 7). The EPM is a highly valid ethological test based on approach-avoidance behavior, widely used to measure anxiety levels [52,53]. The test is based on the tendency of rodents to approach closed areas and avoid open-high areas [39]. Results obtained herein speak in favor of the anxiolytic-like effects of CORM-A1. Additionally, animals with CP/CPPS treated with CORM-A1 made more transitions between light and dark compartments in the LD test with increased time in the light compartment than those treated with PBS (days 3 and 7, Figure 8). Findings in this test also indicate that CORM-A1 treatment decreases anxiety-related behaviors in animals with prostatitis since the LD test is a common anxiety assessment method based on the conflict between instinctive avoidance of bright areas and exploratory behavior [41].

Recent findings from preclinical studies showing the potential of CO to regulate anxiety, like those on traumatic brain injury, hemorrhagic shock, and others are consistent with our finding in the reported behavioral test [54,55,56,57]. Our results are parallel to the studies in which the functional involvement of CO signaling in locus coeruleus has been demonstrated through EPM and LD tests, revealing that it can regulate anxiety levels in rats [56,57]. Actually, CO, either produced endogenously or administered exogenously via slow realizing CO donors, could be implicated in the regulation of anxiety via its effects on the HPA axis, neuroinflammation and redox status, microglial activity, and dopaminergic system.

Prolonged activation of the HPA axis dysfunction contributes to CP/CPPS by increasing the release of proinflammatory cytokines and prostaglandins. This creates oxidative stress and neuroinflammation, leading to cellular damage and aggravating symptoms associated with mental disorders [58]. However, this inflammation and oxidative stress can lead to structural and functional deterioration in the hippocampus. Hippocampal damage weakens the negative feedback mechanism of the HPA axis, causing it to become overactive, which increases the stress response and anxiety levels [59]. In our study, this mechanism may explain the anxiety-related behaviors of the CPPS groups. On the contrary, CORMs are reported to reduce inflammatory responses and protect neuronal cells by increasing heme oxygenase-1 (HO-1) expression in interleukin-1β (IL-1β)-induced neuroinflammation models [60,61]. In line with this information, it can be said that treatment with CORM-A1 in groups with CP/CPPS alleviates anxiety-related behaviors resulting from hippocampal damage that may be caused by oxidative stress. Therewithal, CORM-3 administered intravenously after hemorrhagic shock and resuscitation (HSR) was reported to reduce depression and anxiety-like behaviors, limit damage to the amygdala, and significantly alleviate both behavioral and histopathological deficits by reducing IL-18 levels [62]. Another study showed that the CORM-A1 molecule increased neuronal differentiation, increased the expression of neuronal markers (Nestin, Tuj1, MAP2) and cell proliferation (ki67), and reduced cell death [63]. In addition, it has been reported that CO synthesis in the suprachiasmatic nucleus (SCN) is regulated by cholinergic neurotransmitters and clock gene transcription factors via cGMP signaling pathways and plays a role in balancing anxiety levels [64,65]. Therefore, the observed anxiolytic effect of CORM-A1 in the CP/CPPS model may be due to its regulatory effects on the circadian rhythm in addition to its neuroprotective effect.

Microglial activation has been reported to be triggered by nociceptive stimuli in brain regions associated with emotional responses, such as the hippocampus, prefrontal cortex, and amygdala [66]. This suggests that nociceptive stimuli may lead to mental disorders such as anxiety and depression. On the other hand, at the level of the central nervous system, CO regulates communication between neurons and microglia. Increased HO-1 expression in microglial cells regulates neuroinflammation by increasing endogenous CO production and thus modulates the severity of brain injury [67]. Especially, in vitro studies reveal that CO exhibits neuroprotective effects by increasing the release of neurotrophic factors from microglia cells, while at the same time suppressing inflammatory mediators such as TNF-α and nitrite, and it has also been reported that CO reduces microglial inflammation via Neuroglobin (Ngb) and increases the release of interleukin-10 (IL-10) [68]. Considering the neuroprotective and regulatory effects of CO, it indicates that the inflammatory effects of nociceptive stimuli on microglia can be balanced by the intervention of CO, and that the neurological effects of mental disorders such as anxiety can be alleviated. The anxiolytic effect of CORM-A1 observed in the CP/CPPS model in our study might be explained by the regulatory functions of CO on microglial cells.

Experimental animal studies have reported that exogenous CO administration attenuates hydrogen peroxide (H2O2)-induced cell damage and reduces apoptosis and necrosis. Additionally, overexpression of HO-1 in the dorsal hippocampus region has been shown to produce antidepressant-like effects in behavioral tests such as the forced swim test (FST) and tail suspension test [28]. Although how these mechanisms develop and persist remains a mystery, Bauer and colleagues (2024) have demonstrated that CO has the capacity to regulate both dopamine levels and circadian rhythms, suggesting the existence of the dopamine/HO/CO axis and the neuropsychiatric regulatory potential of CO [69]. Moreover, there are findings that the dopaminergic system plays a critical role in regulating the neurobiological and emotional aspects of pain in CP/CPPS [70,71]. Dopamine release in regions such as the anterior cingulate cortex, mesencephalic dopamine neurons, and nucleus accumbens suppresses the emotional, not perceptual, aspect of pain [72]. Having that in mind, we could assume that regulatory effects of CO on the dopamine/HO/CO signaling axis could be involved in mechanisms of anxiolytic and antinociceptive effects of CORM-A1 in CP/CPPS observed in our current study. In this context, our study suggests that dopamine and the HO/CO axis should be further explored as a common interaction area in the pathogenesis of CP/CPPS, which could provide a basis for further studies examining these signaling pathways at the molecular level.

In this study, we employed a λ-carrageenan-induced CP/CPPS model in rats as an experimental model of CP/CPPS in humans. It produces reproducible pelvic pain, local prostate inflammation, and behavioral alterations resembling clinical CP/CPPS [32,33,34,35,36]. The proven reliability and translational relevance of this model make it particularly suitable for evaluating both analgesic and anxiolytic therapeutic strategies, as employed in the present study.

Several limitations of this study and future research directions could be identified. The administration of CORM-A1 in our study lasted for 7 days, and this period in future research could be longer to determine the full temporal profile of its effects. Also, only a single CORM-A1 dosing regimen (2 mg/kg/day, intraperitoneal) was evaluated, preventing conclusions about dose–response relationships. Although the carrageenan-induced CP/CPPS model has been a reliable model of the human CP/CPPS mosaic, it is also feasible to test the CORM-A1 in other models of CP/CPPS having in mind their complementarity. Behavioral assessments (von Frey, light/dark, open field, and elevated plus maze tests) provide valuable functional insights but are also subject to variability and do not fully capture the multidimensional nature of pain and anxiety in chronic pelvic pain. Finally, the histological analyses were limited to hematoxylin–eosin staining, and more detailed molecular or immunohistochemical assessments could have provided deeper mechanistic understanding. Future studies should consider alternative dosing strategies, longer follow-up, and the incorporation of complementary behavioral and molecular endpoints, as well as validation in other preclinical models, to strengthen translational relevance.

In summary, the effect of exogenous CO on anxiety-related behaviors in CP/CPPS demonstrated its complex regulatory role in the central nervous system. In our study, systemic administration of CORM-A1 significantly ameliorated both mechanical allodynia and anxiety-like behaviors in the CP/CPPS rat model. The findings of our study recommend CO-RMs as potential therapeutic strategies in the management of CP/CPPS. It also traces the road to future studies which should explore varying doses and treatment durations of CORM-A1 to establish optimal therapeutic windows and long-term efficacy. Additional mechanistic investigations using molecular, biochemical, and immunohistochemical analyses are warranted to clarify pathways involved in symptom modulation. Bridging preclinical findings with clinical studies in well-characterized patient cohorts will be essential to determine the relevance of these observations to human CP/CPPS.

## Data Availability

The raw data supporting the conclusions of this article will be made available by the authors on request.

## Abbreviations

CP/CPPS	Chronic Prostatitis/Chronic Pelvic Pain Syndrome
CO-RMs	Carbon Monoxide-Releasing Molecules
OFT	Open Field Test
EPM	Elevated Plus Maze
LD	Light/Dark Box Test
CORM-A1	Carbon Monoxide-Releasing Molecule A1
ROS	Reactive Oxygen Species
HPA axis	Hypothalamic–Pituitary–Adrenal axis
CO	Carbon monoxide
